# Digital motivation for cervical cancer screening: A quasi-experimental trial of the SeDAR Video among middle adulthood Malaysian women

**DOI:** 10.1371/journal.pone.0347489

**Published:** 2026-05-20

**Authors:** Rodziah Romli, Azmawati Mohammed Nawi, Rahana Abd Rahman, Emma Mirza Wati Mohamad, Kah Teik Chew, Syahnaz Mohd Hashim

**Affiliations:** 1 Institut Latihan Kementerian Kesihatan Malaysia (Kejururawatan) Alor Setar, Ministry of Health Malaysia, Alor Setar, Kedah, Malaysia; 2 Department of Public Health Medicine, Faculty of Medicine, Universiti Kebangsaan Malaysia, Cheras, Kuala Lumpur, Malaysia; 3 Department of Obstetrics and Gynecology, Faculty of Medicine, Universiti Kebangsaan Malaysia, Cheras, Kuala Lumpur, Malaysia; 4 Centre for Research in Media and Communication (MENTION), Faculty of Social Sciences and Humanities, Universiti Kebangsaan Malaysia, Bangi, Selangor, Malaysia; 5 Department of Family Medicine, Faculty of Medicine, Universiti Kebangsaan Malaysia, Cheras, Kuala Lumpur, Malaysia; The Chinese University of Hong Kong, HONG KONG

## Abstract

**Background:**

Cervical cancer screening (CCS) is a well-established method for preventing cervical cancer (CC). Electronic health (e-health) interventions have shown potential in disseminating health education and are widely used in developed countries to enhance preventive efforts against CC. This study aimed to evaluate the effectiveness of the electronic health video SeDAR® in increasing CCS uptake, knowledge, and motivational factors related to screening.

**Methods:**

The SeDAR® e-health video was designed based on the Protection Motivation Theory (PMT), incorporating elements such as perceived vulnerability, perceived severity, threat appraisal (fear), coping appraisal, response efficacy, perceived self-efficacy, and protection motivation. It was validated by health experts, media professionals, and female users. A quasi-experimental study was conducted among 76 women, with 38 participants each in the intervention and control groups. The control group received digital pamphlet as intervention tool. Logistic regression was used to assess the impact of SeDAR® on CCS uptake, while repeated measures ANCOVA examined changes in knowledge and motivational constructs.

**Results:**

The attrition rate of participants is 13.2% with the final sample sizes of 35 (7.9%) and 36 (5.3%) in the SeDAR® and control group, respectively. Participants in the SeDAR® intervention group were significantly more likely to undergo CCS (adjusted odds ratio: 7.26, 95% CI: 1.71–30.74, p = 0.007). While no significant difference was observed in knowledge levels between groups, three motivational constructs showed significant changes. Increases in perceived self-efficacy and protection motivation, along with a reduction in threat appraisal (fear), were associated with greater CCS uptake.

**Conclusion:**

The SeDAR® e-health video demonstrates effectiveness as a health promotion tool for enhancing motivation and uptake of cervical cancer screening. It offers a promising, scalable approach to support preventive health behavior among women. The authors confirm that all related trials for this intervention are registered under ClinicalTrials.gov with protocol registration number NCT05426642 and release date on June 20, 2022.

## Introduction

Cervical cancer remains one of the most preventable yet deadly cancers affecting women worldwide. By 2030, global estimates predict over 700,000 new cases and approximately 400,000 deaths annually, with the vast majority occurring in low- and middle-income countries (LMICs) [1]. The age-standardized incidence rate (ASIR) of cervical cancer varies substantially across countries, ranging from less than 2 to over 75 cases per 100,000 women, underscoring persistent global health disparities. The highest incidence rate of cervical cancer, which is more than 26.0 per 100,000 women, is reported in LCMICs on the African continent such as South Africa, Zimbabwe, Mozambique, Zambia, Mali, Senegal and Western Sahara [2]. While the lowest incidence rate of less than 7.0 per 100,000 is reported in developed countries such as the United States, Canada, Australia and developing countries such as Saudi Arabia, Kuwait and Qatar [2]. In Malaysia, the incidence rate reduced from 7.6 to 6.2 per 100 000 populations [3,4] but still remains higher than high-income countries (7.5) per 100 000 [2,4] Alarmingly, more than 85% of cervical cancer cases occur among young, socioeconomically disadvantaged, and less-educated women residing in under-resourced regions [1].

Persistent infection with high-risk strains of the human papillomavirus (HPV) is the primary etiological factor for cervical cancer. Despite its oncogenic potential, HPV infection is frequently asymptomatic in its early stages and may spontaneously resolve within two to three years [5,6]. This asymptomatic nature often results in low risk perception and delayed screening, which are major barriers to early detection and timely treatment [7–9]. Without early screening, up to 90% of HPV infections remain undiagnosed during the critical window for intervention [5].

In Malaysia, despite the implementation of free cervical cancer screening (CCS) services through public healthcare clinics, screening uptake remains suboptimal. Between 2014 and 2018, national coverage ranged from 23% to 26%, and in 2019 only 36.6% of eligible women underwent screening, far below the World Health Organization’s recommended threshold of 70% [3]. Numerous studies in both local and global contexts have identified key barriers to screening, including limited awareness, poor knowledge, low perceived susceptibility, and inadequate engagement by healthcare providers [8,10,11]. Current health promotion efforts often passive and opportunistic, have failed to address these systemic gaps or stimulate behavior change at scale.

Digital health interventions are emerging as promising tools to overcome these challenges, especially in resource-constrained settings. The World Health Organization has advocated for the strategic use of digital platforms to enhance health promotion and service delivery [12,13]. Among these, video-based educational tools have gained traction for their ability to deliver engaging, culturally relevant, and easily accessible health messages. When well-designed, such tools can increase knowledge, modify risk perceptions, and enhance motivation to engage in preventive behaviours [14]. The development of SeDAR e-health involved women’s need which been recommended by WHO [1], is seen as being able to motivate as well as increasing women’s knowledge and practice as compared to the existing MoH pamphlets which are too focused on knowledge aspects only. E-health intervention with digital tools have been found effective in improving cervical cancer screening in previous studies [15–17]. Therefore, the aimed of this study is to test the effectiveness of newly design electronic health SeDAR® in increasing the cervical cancer screening (CCS), knowledge and identify the motivational aspects towards uptake.

## Materials and methods

### Development and validation of e-health SeDAR®

The SeDAR® e-health motivational video was developed based on the Protection Motivation Theory (PMT), incorporating its seven key constructs: perceived vulnerability, perceived severity, threat appraisal (fear), coping appraisal, response efficacy, perceived self-efficacy, and protection motivation. PMT was founded by Roger in 1975 to explain how an individual is motivated to protect themselves from health threats [18,19]. PMT has been widely used in research to improve understanding of health risks and women’s protective attitudes in understanding a health problem [20]. Previous studies have proven the suitability of PMT to be used in framing women’s motivation towards increasing cervical cancer screening rates [21,22].

This study was part of a larger study which performed based on Design and Development Research (DDR). A triangulation approach was employed in its development, incorporating three data sources: (i) a needs analysis via cross-sectional survey (N = 526) [23], (ii) perspectives of health professionals (n = 12) using the Nominal Group Technique (NGT) [24], and (iii) user perspectives through in-depth interviews with women (n = 7) [24].

The final product underwent content validation by health expert (HE) (n = 12) for the scientific material and its accuracy. The media experts (ME) (n = 5) clarified the duration of the video presentation, the method of presentation, and its quality. Whereas the usability of video was verified by end users (n = 11) [25]. The validation yielded a good Content Validity Index (CVI) among the HE (scale-level CVI-average [SCVI/Ave] = 0.986; scale-level CVI-universal agreement [SCVI/UA] = 0.900) and ME (SCVI/Ave = 0.979, SCVI/UA = 0.897) [25]. The usability of newly developed e-health tool SeDAR showed highest Video Engagement Scale (VES®) score [mean (±SD) = 92.90(±3.46)] which indicated the ecological validity of SeDAR® [25].

### Study design

A non-equivalent, quasi-experimental study was conducted entirely through digital platforms to evaluate the effectiveness of the SeDAR® video on cervical cancer screening behavior, knowledge, and motivational constructs. As this study was conducted entirely electronically, contamination between groups could occur if a randomized control trial (RCTs) was used within the same time interval. The use of quasi-experimental is similar to RCTs in many aspects, including the presence of a control group with plausible interventions [26]. However, there are challenges in implementing quasi-experimental study regarding the threats of internal validity that can arise from non-randomization sampling [27]. Since internal validity cannot be overcome through a quasi-experimental, the use of multiple logistic regression and repeated measures ANCOVA statistical analysis is a solution by controlling for potential confounders [28]. Nevertheless, quasi-experiments can still be the most feasible method, with many advantages that have been discussed by many previous scholars [26,27,29]. The authors confirm that all related trials for this intervention are registered under ClinicalTrials.gov with protocol registration number NCT05426642 and release date on June 20, 2022.

### Sample size and setting

The required sample size was calculated using PS Power and Sample Size Calculations software [30], based on the effect size from Abiodun et al. (2014), which examined electronic interventions promoting cervical cancer screening (CCS). A power of 80% was targeted, with an expected 40% attrition rate. A total of 76 women were recruited, with 38 participants each in the SeDAR® intervention and control groups. Participants were recruited voluntarily with a convenience sampling technique via online invitations disseminated throughout WhatsApp groups targeting Malaysian women. Eligibility criteria included: (1) females aged 21–65 years; (2) not having undergone CCS in the past five years; and (3) not having participated in the previous SeDAR® usability study [25]. Women were excluded if they had been diagnosed with cervical cancer or any other reproductive cancers (e.g., cancer of the vulva, vagina, uterus, fallopian tubes, or ovaries), or if they withdrew or failed to complete the follow-up assessments. To ensure group separation and prevent contamination, recruitment for the control and intervention groups occurred at different time points. Participants’ phone numbers and the last six digits of their national identification numbers were cross-checked to avoid duplicate enrolment.

### Instruments

Three tools were used in this study

1. Self-Administered Questionnaire (administered via Google Forms at three time points: pre-intervention, Week 4 post-intervention, and Week 8 post-intervention) was sent to both groups via WhatsApp. The selection of a post-intervention assessment period of two to three months was also implemented in previous studies on the evaluation of CCS uptake [12,17,31]. Therefore, a period of eight weeks was sufficient for the study participants to undertake screening.

The questionnaire comprised

Section 1: Sociodemographic and reproductive health information. The screening uptake is assessed through self-report by stating the date and place of screening as well as the type of screening chosen (Pap smear screening or HPV test). Previous studies conducted in Malaysia also implemented self-report without medical record verification to identify CCS [32].

Section 2: Knowledge on cervical cancer (n = 4, i.e., *cervical cancer usually have no symptoms at the early stages and cause women to be unaware the onset of abnormal changes in cervical cells*) and screening (n = 2, i.e., *Cervical cancer screening can be done with Pap smear test, Cervical cancer screening can also be done by yourself using an HPV test kit obtained from health premises*), assessed using six Likert-scale items (0 = strongly disagree to 10 = strongly agree; total score: 0–60). All items were validated with Content Validity Index among health professionals (n = 7) (CVI = 0.98) and Face Validity Index among women (n = 10) (FVI = 0.99). Reliability tests among women (n = 50) showed a Cronbach’s alpha of 0.741.

Section 3: Motivational constructs, measured using the validated PMT Malay Scale for Cervical Cancer Screening with reliability testing for internal consistency was determined via the confirmatory factor analysis (CFA) among women aged between 21 and 65 years (n = 150). The CFA indicated a good fit for 24 items (i.e., *I worry about having cervical cancer, If I have cervical cancer, my life will change*, *I am afraid of the examination pain*.) The factor loading (range: 0.45–0.98), average variance extracted (range: 0.44–0.90), and composite reliability (range: 0.69–0.97) indicated that the convergent validity for each construct was acceptable [33].

1. SeDAR® E-Health Video: The SeDAR intervention group received a video link via YouTube (https://youtu.be/SbzLFvZa6gY). The SeDAR® e-health motivational video is a 14-minute short film. The storyboard is based on the true story of a cervical cancer (CC) survivor named “Ana” who shares her life story to convince women not to be afraid to undergo CC screening and emphasize the importance of early and regular screening. The SeDAR® video is in Malay with a few simple English words commonly used by Malaysians. The subtitles are in both Malay and English. The video focused on social support to encourage perceived self-efficacy, better quality of life as response efficacy, and undergoing CC screening. Specifically, CC screening should be performed periodically for protection motivation [22].
2. Standardized Pamphlet from the Ministry of Health (MoH) Malaysia: A digital version (PDF) of the official cervical cancer screening pamphlet was sent to both groups via WhatsApp.

All three tools were given to the SeDAR group as intervention tools, while the control group was only received the self-administered questionnaire and the standardized MoH Malaysia pamphlet.

### Data collection

To avoid contamination between groups, the control group was enrolled from 1^st^ January to 30^th^ March 2023, followed by the intervention group from 1^st^ June to 31^th^ August 2023. After obtaining written informed consent via Google form, participants completed the baseline questionnaire and received the MoH pamphlet. The SeDAR® group also received the e-health video. Follow-up data were collected at Weeks 4 and 8 post-intervention. The total study period spanned six months.

### Data analysis

Data from Google Forms were exported to IBM SPSS Statistics Version 25 for analysis. Initial data checks were conducted for duplicates, outliers, and missing values. Normality of continuous variables was assessed using histograms and Shapiro–Wilk tests (*p* < 0.001–0.038). Descriptive statistics, Chi-square tests, and independent t-tests were used to compare baseline characteristics between groups. Logistic regression was employed to assess the effect of the SeDAR® intervention on CCS uptake. Repeated measures ANCOVA was used to evaluate changes in knowledge and PMT constructs over time, adjusting for potential confounding between knowledge and motivational constructs. Confounder selection followed the principle of controlling for variables associated with both exposure and outcome to yield unbiased effect estimates [34].

### Ethical consideration

This study was funded and approved by the Universiti Kebangsaan Malaysia Medical Research Ethics Committee (grant number: FF-2021–499; date of approval: 28 October 2021). The funders had no role in study design, data collection and analysis, decision to publish, or preparation of the manuscript. Informed consent was obtained electronically via Google Forms. Participants were informed about the study’s aims, procedures, risks, and their rights to withdraw at any stage. The study adhered to ethical principles of autonomy, beneficence, and non-maleficence.

## Results

### Participants characteristics

In order to justify the sample size was not underpower, we have performed post-hoc power analysis with 0.987 power detected. With the power of more than 0.8, the sample size was adequate to achieve the desired levels of power [35]. Both groups were statistically comparable at baseline, indicating homogeneity in sociodemographic and reproductive characteristics, as shown in Table 1. No significant differences were observed between the SeDAR® and control groups across all measured variables.

**Table 1:** Respondents’ characteristics (n = 76).

	SeDAR^®^ group, n (%) (*n*=38)	Control group, n (%) (*n*=38)	*X*^2^ stat. (df)^a^	*p* value
**Sociodemographic**				
Age (years)	40.16(7.41)^b^	38.97(7.44)^b^	−0.69(74)^c^	0.805
21 - 35	9(23.7)	13(34.2)		
36 - 50	27(71.1)	22(57.9)		
51 - 65	2(5.3)	3(4.4)		
Ethnicity			0.140(1)	0.709
Malay	35(92.1)	33(86.8)		
Non-Malay	3(7.9)	5(13.2)		
Education Level			0.536(1)	0.464
Secondary/pre-university education	14(36.8)	11(28.9)		
Higher education	24(63.1)	27(71.1)		
Job status			0.461(1)	0.497
Employee	32(84.2)	34(89.5)		
Not employee	6(15.8)	4(10.5)		
Income (MYR)	4062.26(2247.374)^b^	4036.84(2745.062)^b^	0.044(74)^c^	0.138
Marital status			0.140(1)	0.709
Married (with spouse)	33(86.8)	35(92.1)		
Married (without spouse) / Not married	5(13.1)	3(7.9)		
Residency			0.490(1)	0.484
Urban	21(55.3)	24(63.2)		
Rural	17(44.7)	14(36.8)		
Insurance coverage			0.211(1)	0.646
Yes	18(47.4)	20(52.6)		
No	20(52.6)	18(47.4)		
**Reproductive status**				
Sexually active			1.927(1)	0.165
Yes	34(89.5)	37(97.4)		
No	4(10.5)	1(2.6)		
Age at first sexual intercourse (years)	25.89(4.69)^b^	25.45(4.42)^b^	−0.427(74)^c^	0.805
Age at first pregnancy (years)	22.97(10.67)^b^	22.61(10.42)^b^	−0.152(74)^c^	0.879
Total pregnancies (number)	2.58(1.88)^b^	2.84(2.00)^b^	0.589(74)^c^	0.557
Use of contraception			0.396(1)	0.529
Yes	7(18.4)	5(13.2)		
No	31(81.6)	33(86.8)		

^a^Pearson Chi-square, ^b^Mean(standard deviation), ^c^t value, independent t-test

In terms of sociodemographic characteristics, the mean age of participants in the SeDAR® group was 40.16 years (SD = 7.41), compared to 38.97 years (SD = 7.44) in the control group. The majority of participants in both groups were Malay (SeDAR®: 92.1%; Control: 86.8%), employed (SeDAR®: 84.2%; Control: 89.5%), and married (SeDAR®: 86.8%; Control: 92.1%). More than half of the women in both groups had attained higher education (SeDAR®: 63.1%; Control: 71.1%) and resided in urban areas (SeDAR®: 55.3%; Control: 63.2%).

Regarding reproductive health status, most participants were sexually active (SeDAR®: 89.5%; Control: 97.4%) and did not use contraception (SeDAR®: 81.6%; Control: 86.8%). The mean age at first sexual intercourse was 25.89 years (SD = 4.69) in the SeDAR® group and 25.45 years (SD = 4.42) in the control group. The mean age at first pregnancy was 22.97 years (SD = 10.67) in the SeDAR® group and 22.61 years (SD = 10.42) in the control group.

**Fig 1:**
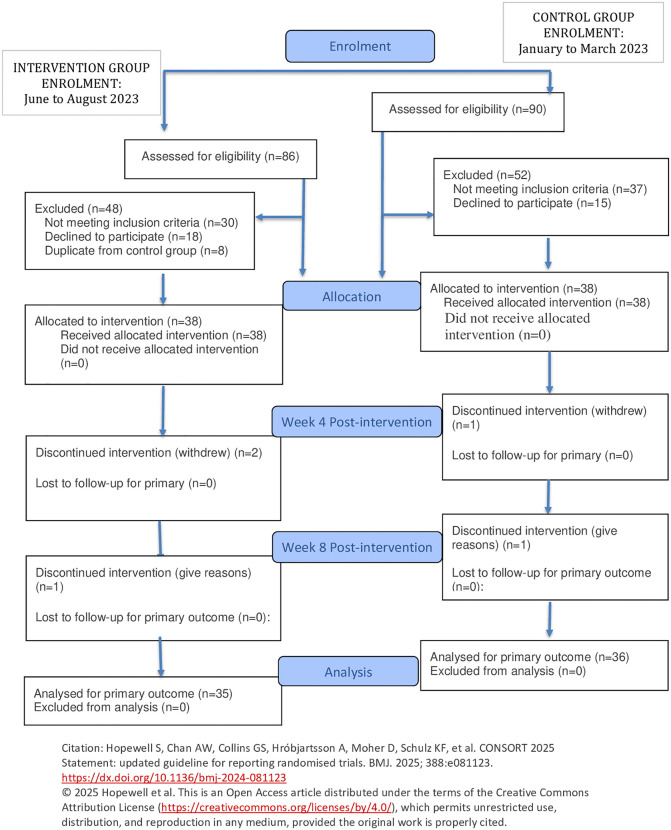
Participant flow chart for quasi-experimental study of the SeDAR® video intervention for cervical cancer screening.

During the first follow-up assessment (Week 4 post-intervention), two participants from the SeDAR® group and one from the control group withdrew from the study. This reduced the sample to 36 participants in the SeDAR® group and 37 in the control group. By the end of the study (Week 8), one additional participant from each group withdrew, resulting in final sample sizes of 35 in the SeDAR® group and 36 in the control group. Fig 1 summarized the flow chart of overall study.

### Effect of e-health motivational video towards CCS

Cervical cancer screening uptake was compared between the SeDAR® and control groups at Weeks 4 and 8 post-intervention. As shown in Table 2, the SeDAR® intervention demonstrated a substantial effect on screening behavior. At Week 8, participants who received the SeDAR® video were significantly more likely to undergo cervical cancer screening compared to those in the control group, with an adjusted uptake ratio of 7.26 (95% CI: 1.71–30.74; p = 0.007), indicating participants in the SeDAR® group were over seven times more likely to undergo screening compared to controls. No statistically significant difference was observed at Week 4.

**Table 2:** The effect of the intervention on the uptake of cervical cancer screening in the fourth and eighth weeks of intervention.

Measurements	Non-adjusted ratio ^a^ [95% CI]	*p-value* ^a^	Adjusted ratio ^b^ [95% CI]	*p-value* ^b^
**Screening uptake at week 4**				
SeDAR^®^ (*n* = 36)	2.11 [0.18–24.43]	0.548		
Control (*n* = 37)	1.00			
**Screening uptake at week 8**				
SeDAR^®^ (*n* = 35)	5.74 [1.46–22.64]	**0.013**	7.26 [1.71–30.74]	**0.007**
Control (*n* = 36)	1.00			

^a^Simple logistic regression

^b^Multiple logistic regression (Adjusted with knowledge scores, perceived vulnerability, perceived severity, threat appraisal (fear), coping appraisal, perceived self-efficacy and protection motivation). Method: Forward/Backword LR. Assumptions were checked and met: no interaction, multicollinearity and outliers detected, Hosmer-Lemeshow Test p = 0.432, specificity 94.6%, sensitivity 40.0%, 83.1% cases were correctly predicted whether or not they had cervical cancer screening at the eighth week of intervention.

***Bold*** is a significant p-value < 0.05

### Effect of e-health motivational video towards knowledge

Table 3 shows the results of the knowledge scores related to cervical cancer and its screening between the SeDAR® and the control group. No significant changes were shown between the groups and the comparison between group*time. However, the effect of time intervention from pre-intervention to the eighth week post-intervention showed a significant increase in knowledge scores (p < 0.001). However, no significant changes were shown when the interaction between group and time was performed. The SeDAR® group did not show a significant difference in the increase in the mean knowledge score despite an increase in scores throughout the intervention period when adjusted for PMT motivation scores [perceived vulnerability, perceived severity, threat appraisal (fear), coping appraisal, perceived self-efficacy and protection motivation]. Therefore, there was no difference in the knowledge score regarding cervical cancer and its screening between the two groups.

**Table 3:** Comparison of mean knowledge scores (intervention effect) in the SeDAR® group and the control group (time effect).

Comparison of knowledge score	Unadjusted mean score [95% CI]	*p*^*a*^ value	Adjusted mean score [95% CI]	*p*^*b*^ value
**Inter-SeDAR**^**©**^ **group**		**0.002** ^**c**^ *		0.926^c^
Pre-intervention	49.23 [46.60–51.86]		49.23 [46.91–51.55]	
Post-intervention week 4	52.06 [48.96–55.16]		52.06 [49.41–54.71]	
Post-intervention week 8	53.34 [50.43–56.25]		53.34 [51.39–55.29]	
**Inter-control group**		0.051^c^		
Pre-intervention	50.50 [48.00–52.99]			
Post-intervention week 4	51.08 [48.74–53.42]			
Post-intervention week 8	53.47 [51.28–55.66]			

^a^Repeated measures ANOVA

^b^*Repeated measures* ANCOVA (Adjusted with perceived vulnerability, perceived severity, threat appraisal (fear), coping appraisal, perceived self-efficacy and protection motivation). Assumptions were reviewed and met: good overall model fit, equal variances, normal distribution.^c^Sphericity Assumed

*The significant inter-intervention time comparison value [pre-intervention and post-intervention week 8, unadjusted mean score difference 4.11 (0.99–7.24), p = 0.007]

Note: Test of effect within SeDAR® group for repeated measures ANOVA: F stat. (df) = 6.97 (2).

**Bold** is a significant p value <0.05

### Effect of e-health motivational video towards motivational constructs (PMT)

Repeated measures ANCOVA was used to evaluate the impact of the SeDAR® intervention on motivational constructs related to cervical cancer screening (CCS), as defined by the Protection Motivation Theory (PMT). These constructs included perceived vulnerability, perceived severity, threat appraisal (fear), coping appraisal, response efficacy, perceived self-efficacy, and protection motivation. The results, presented in Table 4, indicate that there were significant differences in three of the motivational constructs between the SeDAR® and control groups.

**Table 4:** Comparison of mean scores of motivation (intervention effect) adjusted for cervical cancer screening at the eighth week post-intervention between the SeDAR® group and the control group.

Comparison of motivation scores towards cervical cancer screening	Time	Score range	SeDAR® group (n = 35) mean cervical cancer screening (95% CI)		Control group (n = 36) mean cervical cancer screening (95% CI)		Source	F value	*p*^*a*^ value
			Yes (n = 12)	No (n = 23)	Yes (n = 3)	No (n = 33)			
Threat appraisal (fear),	Pre-intervention	3-15	9.3.3 [7.14–11.52]	10.18 [8.63–11.73]	11.52 [7.31–14.74]	8.77	Group*Screening	4.25	**0.043**▪
	Post-intervention week 4		8.76 [6.56–10.96]	10.04 [8.48–11.59]	12.74 [8.51–14.97]	8.87			
	Post-intervention week 8		6.65 [4.46–8.85]	9.92 [8.37–11.48]	9.58 [5.36–13.81]	8.43 [7.17–9.69]			
Perceived self-efficacy	Pre-intervention	6-30	19.45 [15.82–23.09]	19.76 [17.18–22.33]	17.12 [10.12–24.11]	21.93 [19.84–24.03]	Group*Screening	4.67	**0.034**▪▪
	Post-intervention week 4		24.08 [20.88–27.28]	22.39 [20.12–24.65]	20.09 [13.92–26.25]	23.42 [21.58–25.26]			
	Post-intervention week 8		25.59 [23.01–28.18]	22.82 [20.99–24.65]	17.42 [12.44–22.41]	24.39 [22.89–25.88]			
Protection motivation	Pre-intervention	3-15	13.62 [12.00–14.23]	11.73 [10.58–12.87]	10.09 [6.99–13.19]	12.99 [12.06–13.92]	Group*Screening	9.09	**0.004**▪▪▪
	Post-intervention week 4		13.77 [12.56–14.57]	12.39 [11.54–13.25]	10.66 [8.34–12.99]	13.29 [12.59–13.99]			
	Post-intervention week 8		14.32 [13.14–14.89]	12.62 [11.79–13.46]	12.31 [10.05–13.57]	13.24 [12.56–13.92]			

^a^Repeated measures ANCOVA (Adjusted with knowledge score = 53.41). Assumptions were checked and met: good overall model fit, equal variances, normal distribution.

▪The significant inter-intervention time comparison value [post-intervention week 4 and post-intervention week 8, adjusted mean score difference 1.46 (0.10–2.81), p = 0.031]

▪▪The significant inter-intervention time comparison value [pre-intervention and post-intervention week 4, adjusted mean score difference 2.93 (0.62–5.24), p = 0.008]; [pre-intervention and post-intervention week 8, adjusted mean score difference 2.99 (0.47–5.51), p = 0.015]

▪▪▪The significant inter-intervention time comparison value [pre-intervention and post-intervention week 8, adjusted mean score difference 1.02 (0.01–2.04), p = 0.049]

**Bold** is a significant p value <0.05

Specifically, participants in the SeDAR® group demonstrated a significantly lower level of threat appraisal (fear) compared to the control group, with an adjusted mean score difference of 1.46 (95% CI: 0.10–2.81; p = 0.031). They also showed a significantly higher level of perceived self-efficacy, with an adjusted mean score difference of 2.99 (95% CI: 0.47–5.51; p = 0.015). Additionally, protection motivation was significantly higher in the SeDAR® group, with an adjusted mean score difference of 1.02 (95% CI: 0.01–2.04; p = 0.049). These findings suggest that the SeDAR® video intervention was effective in enhancing key motivational factors that influence CCS behavior, particularly by reducing fear and increasing confidence and motivation to participate in screening.

### Barriers towards cervical cancer screening among SeDAR® group

All participants in SeDAR group reported the completion of viewing video and researcher confirm by the link viewed with no technical barrier reported. Among participants in the SeDAR® group who did not undergo cervical cancer screening (n = 23), the main barriers reported were family commitments (39.1%) and anxiety about the screening procedure (21.7%). In addition, a smaller proportion of participants cited fear of pain (13.0%) and feelings of embarrassment (13.0%) as deterrents. Other reported barriers included inconvenient clinic hours (8.0%) and a lack of information about the screening procedure (4.3%). These findings highlight the need to address both emotional and logistical factors to improve screening uptake.

## Discussion

The effectiveness of e-health tools in increasing cervical cancer screening (CCS) uptake has been well-documented in previous studies [15–17]. Video-based interventions, in particular, have consistently demonstrated the ability to significantly encourage women to undergo screening, regardless of study design, type of control group, screening method, or outcome measurement interval [15–17]. In the current study, participants who received the SeDAR® motivational video were nearly seven times more likely to undergo CCS by the eighth week post-intervention, highlighting the strong influence of theory-based digital content on behavior change. This finding aligns with a recent meta-analysis of six e-health interventions, which found that women exposed to digital content were approximately twice as likely to undergo or intend to undergo screening [36].

Given that this study was conducted entirely online, screening uptake was measured through self-report, a common method used in digital intervention studies. This approach is supported by prior work such as Drokow et al. (2021) and Abiodun et al. (2014), who also used self-reported outcomes in quasi-experimental designs. While self-report data may lack the accuracy of medical or administrative records [37], a systematic review by [38] found that most studies assessing the accuracy of self-reported screening data were of fair quality, with acceptable validity. In the context of digital health interventions aiming to reach underserved and geographically dispersed populations, self-report remains a practical and appropriate measure, especially when access to clinical data is limited. While the reliance on self-reported screening uptake is common in digital interventions, the risk of recall or social desirability bias remains. Where resources and institutional support are available, future studies incorporating cytology results could provide more robust validation of screening outcomes by propose medical-record validation in future studies.

Although screening uptake was significantly higher in the intervention group, no significant difference in knowledge scores was observed between the SeDAR® and control groups. This result may be attributed to a ceiling effect, as baseline knowledge scores were already high in both groups, 49.18 (SD = 7.37) in the SeDAR® group and 50.73 (SD = 7.33) in the control group, out of a maximum score of 60. Furthermore, both groups received the same standardized knowledge content via an online PDF leaflet, which may have minimized the differences in knowledge acquisition. The design of the SeDAR® video focused primarily on motivational constructs rather than detailed factual knowledge, which may explain why changes in knowledge were not significantly different despite increased screening behavior.

Importantly, the SeDAR® intervention was effective in modifying key motivational constructs as defined by the Protection Motivation Theory (PMT). Participants in the intervention group reported significantly lower levels of threat appraisal (fear), and significantly higher perceived self-efficacy and protection motivation compared to the control group. These results align with previous studies that found women who undergo CCS typically report less fear and greater confidence in managing the screening process [23]. The motivational video appeared to reduce anxiety associated with receiving a diagnosis or experiencing discomfort during the procedure, two common emotional barriers to screening. Similar findings were observed by Ornelas et al. (2018), who used culturally tailored, entertainment-based video narratives to increase screening intent. When e-health content addresses real-world fears and misconceptions, it can serve as a powerful tool for behavior change.

In addition, the study explored barriers among participants who did not undergo screening. The most commonly cited obstacles were family and work commitments, reported by 39.1% and 21.7% of participants, respectively. These barriers are consistent with findings from earlier Malaysian studies [39,40], which emphasize the time constraints women face in attending in-person screenings. Other barriers, such as fear of pain, embarrassment, clinic operating hours, and lack of procedural information, were reported by fewer participants but remain important considerations for future interventions. Previous studies that allowed longer follow-up periods, such as six months [15] or up to 15 months [16,41] demonstrated higher CCS uptake, suggesting that behavior change may take more time when external barriers are present. While high scores in motivational constructs like self-efficacy and response efficacy were observed, some residual fears persisted, indicating that fear can serve as both a barrier and a driver of action depending on its intensity and how it is addressed [22].

Electronic health promotion plays a role in reinforcing positive health behaviors, especially among the younger generation [42]. The world is moving towards the World Health Organization (WHO) Shanghai Declaration’s decade-long commitment to promote health in the 2030 Agenda by fully utilizing social innovation and interactive technologies to empower health policies [43]. In fact, e-health has been a key focus of WHO since 2005, followed by a resurgence in the use of health-focused technologies by developed countries starting in 2006 [44,45]. Women in the SeDAR® intervention group also gave positive views, where they felt that electronic videos were useful, interactive, and easy to disseminate. The videos were seen as a motivational tool for cervical cancer screening to end the dilemma of fear of screening experienced by women. However, digital divide barriers may exist among LMIC countries such as Malaysia especially in rural area [46,47]. As evidenced in this study, the sample was highly educated, employed, and predominantly urban Malay women who were more e-literate. The e-health approach in providing information without requiring women to attend a health facility has been proven through this study where women have been proven to receive a motivational boost to undergo CC screening after being given an intervention through the SeDAR® motivational tool via online.

## Conclusion

The SeDAR® e-health video demonstrates effectiveness as a health promotion tool for enhancing motivation and uptake of cervical cancer screening. Increases in perceived self-efficacy and protection motivation, along with a reduction in threat appraisal (fear), were associated with greater CCS uptake. The strength of this study is that its culturally tailored content makes it particularly relevant not only for Malaysian women but also for audiences across Southeast Asian countries with similar cultural backgrounds. Besides, the SeDAR® video serves as a form of educational entertainment, delivering motivational messaging through an accessible and engaging format. Its use is recommended for health professionals as part of digital health education strategies, and for women as a motivational resource to support informed decision-making about cervical cancer screening. The limitation of study is related to self-reported screening. Since this study was conducted entirely electronically and no screening services were offered, self-reported screening may be questionable. Moreover, the non-concurrent recruitment among study and control group may introduce a potent unaddressed confounder such as external public health campaigns or policy changes occurring between recruitment periods. Besides, current finding could not generalize the eligible women who are from rural area, low-literacy and have boundaries for internet access. Future studies may be able to verify screening through medical reports and access women from rural areas, low-literacy and have boundaries for internet access.

## Supporting information

S1 File Study protocol UKM. This is the protocol submission for UKM ethical approval.
(PDF)
