# Supplementary material for: Digital motivation for cervical cancer screening: A quasi-experimental trial of the SeDAR Video among middle adulthood Malaysian women
Source: PLoS One. 2026 May 20;21(5):e0347489. doi: 10.1371/journal.pone.0347489 (PMC13189345; doi:10.1371/journal.pone.0347489)
Supplement: S1 File — This is the protocol submission for UKM ethical approval. (PDF) [file pone.0347489.s001.pdf]

1. APPLICANT (PRINCIPAL INVESTIGATOR)
APPLICANT (PRINCIPLE INVESTIGATOR)

A.	**NAME**<br>*NAME*	Prof Madya Dr Azmawati Mohammed Nawi
B.	**UKM PER**<br>*ID NO.*	K011169
C.	**DEPARTMENT**<br>*DEPARTMENT*	Department of Community Health
D.	**EMAIL**<br>*EMAIL*	azmawati@ppukm.ukm.edu.my
E.	**MOBILE NUMBER**<br>*MOBILE NUMBER*	019-3131340

2. RESEARCH PROJECT: (Summary of research proposal)
RESEARCH PROJECT: (Summary of proposal)

Cervical cancer remains the third leading cause of death among women in
Malaysia although globally it is known that early cervical cancer screening is an effective preventive measure. The majority of cervical cancer cases are diagnosed at a late stage and contribute to low survival rates as well as difficult disease management. Previous studies have shown that limited exposure to information related to cervical cancer and its screening is associated with poor knowledge, indifferent attitudes, low motivation, and low screening practices among women. Health education interventions have successfully improved women's knowledge, attitudes and practices regarding cervical cancer and its screening. However, the researcher believes that conservative and opportunistic intervention approaches are less able to reach marginalized women who have limited access to health facilities.

The development of an electronic video health education module that is easily accessible without requiring women to attend health facilities should be planned. Based on the study justification outlined, the researcher will design and develop the Cervical Cancer Module; E-Education for Cultivating Women's Lifelong Screening Routine (KaSEH) in the form of an e-education video based on women users' knowledge and motivation while meeting the informational needs of health professional groups as information providers. This module will become a health education tool that can be used through various electronic technology platforms, mass media, displays at health facilities, and information dissemination through mobile phones. The accessibility and ease of dissemination of the KaSEH Module video are expected to contribute to improved women's knowledge, motivation and practices regarding cervical cancer and its screening.

This study will use the Design & Development Research (DDR) approach with multiple research methods, namely quantitative study, quasi-experimental study, Nominal Group Technique (NGT), and Fuzzy Delphi Method (FDM). There are three research phases: Phase 1, the researcher will obtain module needs analysis information from prospective module users, namely women; Phase 2, the process of designing and developing the KaSEH Module video; and Phase 3, evaluation of the effectiveness of the KaSEH Module video. Findings from all DDR phases will answer the research questions and objectives to achieve the final research goal, namely the production of the KaSEH Module video to improve women's motivation regarding cervical cancer and its screening.

A	**TITLE :**<br>Development and Evaluation of the KaSEH Video Module to Improve Women's Knowledge and Motivation Regarding Cervical Cancer and Its Screening.
B	**KEYWORDS :**<br>e-education video, women's motivation, cervical cancer, lifelong screening
C	**DURATION: (not more than 3 years - except for clinical research projects)**<br>*DURATION: (not more than 3 years - except for clinical trial research project)*<br><br>Duration : _______36________ (month(s))<br>*Duration (month(s))*<br><br>Date of commencement : ____20/09/21_____ (dd/mm/yy)<br>*Date of commencement (dd/mm/yy)*<br><br>Date of completion : ___ 19/09/24_____ (dd/mm/yy)<br>*Date of completion (dd/mm/yy)*

D	**CATEGORY OF RESEARCH PROJECT**<br>*CATEGORY OF RESEARCH PROJECT*:<br><br>[ ]Bachelor Degree Degree<br>[ ]Doctor of Medicine Special Study Module<br>[ ]Master Masters<br>[/]PhD *PhD*<br>[ ]DrPH *DrPH*<br>[ ]Lecturer Lecturer<br>[ ]Sabbatical Leave Sabbatical Leave<br>[ ]Practicum Practicum<br>[ ]Others (Please state) Others (Please state) …………………………………………………
E	**TYPE OF RESEARCH PROJECT**<br>*TYPE OF RESEARCH PROJECT*:<br><br>[/]Clinical Trial Clinical Trial<br>[ ]Cohort Cohort<br>[ ]Case control Case control<br>[/]Cross sectional Cross sectional<br>[ ]Systematic review Systematic review<br>[ ]Laboratory/cell study Experimental design

F	**RESEARCH CLUSTER :**<br>*RESEARCH CLUSTER :*<br>*Refer to Attachment A. Please refer to the Guidelines for Completing the Research Approval Application Form<br><br>Advanced Health and Medicine<br><br>**mandatory/required *Refer Attachment A
G	**RESEARCH AREA :**<br>*RESEARCH AREA :*<br>*Refer to Attachment A. Please refer to the Guidelines for Completing the Research Approval Application Form<br><br>Medical and Health Science<br><br>**mandatory/required *Refer Attachment A
H	**RESEARCH LEVEL :**<br>*RESEARCH LEVEL :*<br>[/]University University<br>[ ]National National<br>[ ]International International
I	**RESEARCH TYPE :**<br>*RESEARCH TYPE:*<br>[ ]Fundamental Fundamental<br>[/]Applied Applied (Problem Solving)
J	**COMMERCIAL STATUS :**<br>*COMMERCIAL STATUS:*<br><br>[ ]Has Been Commercialized Has Been Commercialized<br>[ ]In The Commercialization Process In The Commercialization Process<br>[/]Potential For Commercialization Potential for Commercialization<br>[ ]Unsure Unsure<br>[ ]Cannot Be Commercialized/Not Applicable Cannot Be Commercialized/Not Applicable

K **EXPECTED OUTPUT :**

EXPECTED OUTPUT:

Number of *Doctor of Philosophy / Master students : 1

Number of PhD / Master Student:

Number of *Research Assistants / Graduate Research Assistants (GRA) / Postdoctoral Researchers : -

Number of Research Assistant / Graduated Research Assistant / Post-Doc Research:

Number of *Journals / Proceedings / Books : 2

*Number of *Journal / Proceedings / Book:*

Number of *Intellectual Property / Commercialisation / Licensed Technology : 1

Number of Intellectual Property / Commercial / Technology Licensing:

Network & Collaboration: * National / International: 1

*Network & Collaboration: * National / International*

L **GANTT CHART (RESEARCH ACTIVITIES) :**

(Activities begin with data collection. The start date and end date must be the same as the duration stated in Section C)

GANTT CHART (RESEARCH ACTIVITIES):

(Activity starts with data collection. The start date and the end date shall be the same as the period specified in Part C)

**RESEARCH ACTIVITIES**<br>*RESEARCH ACTIVITIES*	**START DATE**<br>*START DATE*	**END DATE**<br>*END DATE*
Validation of questionnaire form	20.09.21	31.01.22
Phase 1: Module needs analysis	01.02.22	30.06.22
Phase 2: Module design	01.07.22	31.12.22
Phase 2: Module development	01.01.23	31.08.23
Phase 3: Evaluation of module effectiveness	01.09.23	30.02.24
Data analysis	01.03.24	30.06.24
Penulisan dan penghantaran manuskrip & laporan akhir	01.07.23	19.09.24

M **MILESTONE :**
(Milestones or achievements every 6 months must correspond to the activities stated in Section K)
MILESTONE:
(Milestone or achievement every 6 months shall correspond to the activities specified in Section K)

**JEJAK KUNCI**<br>*Milestone*	**TARIKH DIJANGKA SIAP**<br>*EXPECTED DATE OF COMPLETION*
Validation of questionnaire form	31.01.22
Phase 1: Module needs analysis	30.06.22
Phase 2: Module design	31.12.22
Phase 2: Module development	31.08.23
Phase 3: Evaluation of module effectiveness	30.02.24
Data analysis	30.06.24
Penulisan dan penghantaran manuskrip & laporan akhir	19.09.24

N **JANGKAAN TAHAP RISIKO :**
ESTIMATE THE DEGREE OF RISK :

**Risiko**	**Rendah**	**Sederhana**	**Tinggi**	**Catatan penilaian risiko**<br>*Nyatakan faktor-faktor yang boleh menyebabkan kelewatan, atau menghalang pelaksanaan projek.*
**Technical**		/		- Proses penghasilan video mungkin akan mengalami kesulitan teknikal akibat pandemik COVID-19
**Time**		/		- Pengumpulan pakar untuk tarikh perlaksanaan NGT<br>- Penghasilan video lewat akibat pendemik Covid-19
**Kewangan/ Bajet**		/		- Bajet penghasilan video yang mungkin meningkat<br>- Bajet Honorarium

3. CO-RESEARCHER INFORMATION

(perakuan adalah tertakluk dengan persetujuan pada syarat-syarat pada polisi penerbitan di Lampiran D. Sila rujuk Garis Panduan Mengisi Borang Application Kelulusan Menjalankan Penyelidikan)

CO-RESEACHER(S) INFORMATION

(Endorsement is subject to agreement with the terms stating in the publication policy in Attachment D. Please refer to Guideline for Fill out the Research Application Form)

**PENYELIDIK UKM**<br>*UKM RESEARCHERS*				
**Bil.**<br>*No.*	**Nama & Emel**<br>*Name & Email*	**UKM Per**<br>*ID No.*	**Jabatan**<br>*Department*	**Signature**<br>*(Digital signature is accepted)*<br>*Signature*
1	**Nama** *(Name)* :<br>Assoc. Prof. Dr Rahana Abd Rahman<br>**Emel** *(Email)* :<br>drrahana@ppukm.ukm.edu.my	K013430	Department of Obstetrics & Gynaecology	
2	**Nama** *(Name)* :<br>Assoc. Prof. Dr Emma Mirza Wati Mohamad<br>**Emel** *(Email)* :<br>emmamohamad@ukm.edu.my	K013922	Centre for Research in Media and Communication (MENTION)	
3	**Nama** *(Name)* :<br>Dr Syahnaz Mohd Hashim<br>**Emel** *(Email)* :<br>syahnaz@ppukm.ukm.edu.my	K013772	Department of Family Medicine	
4	**Nama (Name) :**<br>Dr Chew Kah Teik<br>**Emel (Email) :**<br>drchewkt@gmail.com	K020145	Department of Obstetrics & Gynaecology	

**PENYELIDIK LUAR**<br>*OUTSIDE RESEARCHERS*				
**Bil.**<br>*No.*	**Nama**<br>*Name*	**No. Kad Pengenalan/ Pasport**<br>*IC No./Passport No.*	**Organisasi** *Organization*	**Signature**<br>*(Digital signature is accepted)*<br>*Signature*
	**Nama (Name) : NONE**<br>**Emel** *(Email)* **:**			

PELAJAR<br>*STUDENT*				
**Bil.**<br>*No.*	**Nama**<br>*Name*	**No. Kad Pengenalan/ Pasport**<br>*IC No./Passport No.*	**Jabatan / Organisasi**<br>*Department /Organization*	**Signature**<br>*(Digital signature is accepted)*<br>*Signature*
1	**Nama** *(Name)* :<br>Rodziah binti Romli<br>**Emel** *(Email)* :<br>P109869@siswa.ukm.edu.my	840608-02-5364	Institut Latihan KKM (Pembantu Perubatan) Alor Setar, Kedah	

*Please add rows to the table if space is insufficient
* *Please insert rows to the table if space is insufficient*

4. EXTERNAL GRANT RECEIVED
EXTERNAL GRANT RECEIVED

***If external funding is received, the applicant does not need to complete the section "FINANCIAL REQUIREMENTS AND JUSTIFICATION FOR THE RESEARCH PROJECT" in this form.**
Please leave out the page on "FINANCIAL ESTIMATION AND JUSTIFICATIONS FOR RESEARCH PROJECT" if this research is sponsored externally.

**Geran Luar Yang Diterima**<br>*External Grant Received*	**Jumlah (RM)**<br>*Total (RM)*

Jumlah: ……………………………………………………..
Total:

**PLEASE ATTACH A CONFIRMATION LETTER FROM THE SPONSORING COMPANY
*******Please enclosed endorsement letter from company*

***** Please refer to Attachment B**
*** *Please refer to Attachment B*

5. FINANCIAL REQUIREMENTS AND JUSTIFICATION FOR THE RESEARCH PROJECT
FINANCIAL ESTIMATION AND JUSTIFICATION FOR RESEARCH PROJECT

(Not required if external funding is received)
(Not applicable to externally sponsored research)

PERKARA UTAMA<br>*MAIN ITEM*	JUSTIFIKASI<br>*JUSTIFICATION*	AMAUN/KUANTITI<br>*AMOUNT/QUANTITY*	JUMLAH (RM)<br>*TOTAL (RM)*	JUMLAH DILULUSKAN (RM)<br>*TOTAL APPROVED (RM)*
**VOTE21000: PECAHAN BELANJAWAN: PERJALANAN DAN PENGANGKUTAN**<br>*VOTE21000: PORTION OF BUDGET: TRAVELLING AND TRANSPORTATION*				
Penghasilan modul Video	Return transportation during the production process of the e-education video module.	RM190	RM190	KEGUNAAN PEJABAT<br>*FOR OFFICE USE*
**VOTE 27000: PECAHAN BELANJAWAN: BAHAN & BEKALAN PENYELIDIKAN**<br>*VOTE 27000: PORTION OF BUDGET: RESEARCH MATERIAL & SUPPLY*				
-				
**VOTE 29000: PERKHIDMATAN IKHTISAS**<br>*VOTE 29000: PROFESSIONAL SERVICE*				
Honorarium for study respondents	-Phase 1:<br>206 (women) x RM 10 =<br><br>-Phase 2:<br>8 (women NGT) x RM50 =<br>12 (NGT experts) x RM100 =<br>12 (FDM 1 experts) x RM100 =<br>12 (FDM 2 experts) x RM100 =<br><br>-Phase 3:<br>30 (pilot) x RM10 =<br>60 (quasi-experiment) x RM10	 RM2060<br><br><br>RM400<br>RM1200<br>RM1200<br>RM1200<br><br><br>RM300<br>RM600	RM29 810	
Honorarium payment for video developer	Penghasilan modul video ependidikan menggunakan khidmat teknologi	RM22 850 (Lampiran)		
**VOTE 11000: UPAH DAN ELAUN** *VOTE 11000 : SALARY AND ALLOWANCE*				
-				
**VOTE 24000: SEWAAN**<br>*VOTE 24000: RENTAL*				
-				

*Refer to Attachment C
**Refer Attachment C*

Attachment: Quotation for Technology Services

6. PERAKUAN PEMOHON:

(Endorsement is subject to agreement with the terms and conditions in the publication policy in Attachment D. Please refer to the Guidelines for Completing the Research Approval Application Form)

APPLICANT ENDORSEMENT:

(Endorsement is subject to agreement with the terms stating in the publication policy in Attachment D. Please refer to Guideline for Fill out the Research Application Form)

……………………………………………….. …………16/8/2021……………………………

Signature Pemohon **Date**

Applicant Signature *Date*

7. RECOMMENDATION BY HEAD OF DEPARTMENT:

RECOMMENDATION BY HEAD OF DEPARTMENT:

[ ] This application has been presented, amended and approved at the department level / (for student projects only).

This application has been presented, corrected and approved at the department level (student project only).

(Please leave this section blank if the applicant is the Head of Department)

(Please leave this column blank if the applicant is the Head of Department)

Application supported

……..

……..

……………………………………………….. …………16/8/2021…………

Signature **Date**

Signature *Date*

8. DECISION OF FACULTY RESEARCH COMMITTEE

DECISION OF FACULTY RESEARCH COMMITTEE:

……..

……..

Approved budget: RM …………………………………………

Approved Budget:

……………………………………………….. ……………………………………….

Signature **Date Signature**

Date
